# Validation of the PANAMA Score for Survival and Benefit of Adjuvant Therapy in Patients With Resected Pancreatic Cancer after Neoadjuvant FOLFIRINOX

**DOI:** 10.1097/SLA.0000000000006650

**Published:** 2025-01-31

**Authors:** Ingmar F. Rompen, Thomas F. Stoop, Stijn van Roessel, Eran van Veldhuisen, Quisette P. Janssen, Adnan Alseidi, Alberto Balduzzi, Gianpaolo Balzano, Frederik Berrevoet, Morgan Bonds, Olivier R. Busch, Giovanni Butturini, Ammar A Javed, Marco Del Chiaro, Kevin C. Conlon, Massimo Falconi, Isabella Frigerio, Giuseppe K. Fusai, Johan Gagnière, Oonagh Griffin, Thilo Hackert, Ernesto Sparrelid, Asif Halimi, Knut J. Labori, Giuseppe Malleo, Marco V. Marino, Michael B. Mortensen, Andrej Nikov, Mickaël Lesurtel, Tobias Keck, Jörg Kleeff, Rupaly Pandé, Per Pfeiffer, Daniel Pietrasz, Keith J. Roberts, Antonio Sa Cunha, Roberto Salvia, Oliver Strobel, Timo Tarvainen, Hanneke W. M. van Laarhoven, Bas Groot Koerkamp, Martin Loos, Christoph W.﻿ Michalski, Marc G. Besselink, Thomas Hank

**Affiliations:** * Department of General, Visceral and Transplantation Surgery, Heidelberg University Hospital, Heidelberg, Germany; † Department of Surgery, Amsterdam UMC, location University of Amsterdam, Amsterdam, The Netherlands; ‡ Cancer Center Amsterdam, Amsterdam, The Netherlands; § Department of Surgery, Erasmus MC Cancer Center, Rotterdam, The Netherlands; ∥ Department of Surgery, University of California at San Francisco, San Francisco; ¶ Department of General and Pancreatic Surgery, The Pancreas Institute, University of Verona Hospital Trust, Verona, Italy; # Department of Medicine and Technological Innovation (DIMIT), Pancreas Unit, Ospedale di Circolo, Insubria University, Varese, Italy; ** Department of General and HPB Surgery and Liver Transplantation, Ghent University Hospital, Ghent, Belgium; †† Department of Surgery, Oklahoma University Health Science Center, Oklahoma City, Oklahoma; ‡‡ Department of Surgery, Pederzoli Hospital, Peschiera, Italy; §§ Department of Surgery, University of Colorado Hospital, Aurora; ∥∥ Department of Surgery, Trinity College Dublin, Trinity Centre for Health Sciences, Dublin, Ireland; ¶¶ Department of Pancreatic Surgery, IRCCS San Raffaele Hospital, Vita-Salute University, Milano, Italy; ## Collegium Medicum, University of Social Sciences, Lodz, Poland; *** Hepatobiliary Surgery and Liver Transplantation Unit, Royal Free Hospital, London, UK; ††† Department of Digestive and Hepatobiliary Surgery-Liver Transplantation, University Hospital of Clermont-Ferrand, Clermont-Ferrand, France; ‡‡‡ National Surgical Centre for Pancreatic Cancer, St Vincent’s University Hospital, Dublin, Ireland; §§§ Department of General, Visceral and Thoracic Surgery, University Hospital Hamburg-Eppendorf, Hamburg, Germany; ∥∥∥ Division of Surgery and Oncology, Department of Clinical Science, Intervention and Technology, Karolinska Institutet, Karolinska University Hospital, Stockholm, Sweden; ¶¶¶ Department of Diagnostics and Intervention, Surgery, Umeå University, Umeå, Sweden; ### Department of Hepato-Pancreato-Biliary Surgery, Oslo University Hospital, Oslo, Norway and Institute of Clinical Medicine, University of Oslo, Oslo, Norway; **** Department of General Surgery, Azienda Ospedaliera Ospedali Riuniti Villa Sofia-Cervello, Palermo, Italy and Oncologic Surgery Department, P. Giaccone University Hospital, Palermo, Italy; †††† Department of Surgery, Odense Pancreas Center, Odense University Hospital, Odense, Denmark; ‡‡‡‡ Department of Surgery, Charles University and Central Military Hospital, Prague, Czech Republic; §§§§ Department of Digestive Surgery and Liver Transplantation, Croix Rousse University Hospital, Hospices Civils de Lyon, University of Lyon, Lyon, France; ∥∥∥∥ Department of Surgery, University Medical Center Schleswig-Holstein UKSH, Campus Luebeck, Luebeck, Germany; ¶¶¶¶ Department of Visceral, Vascular and Endocrine Surgery, Martin Luther University Halle-Wittenberg, Halle, Germany; #### Department of Surgery, University Hospital Birmingham, Birmingham, UK; ***** Department of Medical Oncology, Odense University Hospital, Odense, Denmark; ††††† Department of Hepato-Biliary-Pancreatic Surgery, Liver Transplant Center, Paul Brousse Hospital, Université Paris-Sud, Université Paris-Saclay, Villejuif, France; ‡‡‡‡‡ Department of General Surgery, Division of Visceral Surgery, Medical University of Vienna, Vienna, Austria; §§§§§ Department of Gastroenterological Surgery, Helsinki University Hospital, Helsinki, Finland; ∥∥∥∥∥ Department of Medical Oncology, Amsterdam UMC, location University of Amsterdam, Amsterdam, The Netherlands

**Keywords:** adjuvant chemotherapy, FOLFIRINOX, neoadjuvant treatment, pancreatic neoplasm, prognostic score

## Abstract

**Objective:**

To validate the prognostic value of the PAncreatic NeoAdjuvant MAssachusetts (PANAMA) score and to determine its predictive ability for survival benefit derived from adjuvant treatment in patients after resection of pancreatic ductal adenocarcinoma (PDAC) following neoadjuvant FOLFIRINOX.

**Background:**

The PANAMA score was developed to guide prognostication in patients after neoadjuvant therapy and resection for PDAC. As this score focuses on the risk for residual disease after resection, it might also be able to select patients who benefit from adjuvant after neoadjuvant therapy.

**Methods:**

This retrospective international multicenter study is endorsed by the European-African Hepato-Pancreato-Biliary Association. Patients with PDAC who underwent resection after neoadjuvant FOLFIRINOX were included. Mantel-Cox regression with interaction analysis was performed to assess the impact of adjuvant chemotherapy.

**Results:**

Overall, 383 patients after resection of PDAC following neoadjuvant FOLFIRINOX were included of whom 187 (49%), 137 (36%), and 59 (15%) had a low-risk, intermediate-risk, and high-risk PANAMA-score, respectively. Discrimination in median overall survival (OS) was observed stratified by risk groups (48.5, 27.6, and 22.3 months, log-rank *P*_low-intermediate_ = 0.004, log-rank *P*_intermediate-high_ = 0.027). Adjuvant therapy was not associated with an OS difference in the low-risk group [hazard ratio (HR): 1.50, 95% CI: 0.92–2.50], whereas improved OS was observed in the intermediate (HR: 0.58, 95% CI: 0.34–0.97) and high-risk groups (HR: 0.47, 95% CI: 0.24–0.94; *P* interaction = 0.008).

**Conclusions:**

The PANAMA 3-tier risk groups (low-risk, intermediate-risk, and high-risk, available through pancreascalculator.com) correspond with differential survival in patients with resected PDAC following neoadjuvant FOLFIRINOX. The risk groups also differentiate between survival benefits associated with adjuvant treatment, with only the intermediate- and high-risk groups associated with improved OS.

There has been a paradigm shift in the management of patients with borderline resectable pancreatic ductal adenocarcinoma (PDAC) towards neoadjuvant treatment with increased use also being observed in the resectable staged patients.^1–3^ Furthermore, induction chemotherapy is used in patients with locally advanced PDAC with the goal of obtaining a radical resection and selecting appropriate candidates.^1–3^

After neoadjuvant/induction chemotherapy (hereafter: summarized as neoadjuvant treatment), classic pathologic determinants change through the course of cytotoxic treatment and may lose their prognostic value.^4–7^ The PAncreatic NeoAdjuvant MAssachusetts (PANAMA) score was created as a specific prognostication score after neoadjuvant treatment, incorporating CA19-9 after neoadjuvant therapy and margin status in addition to residual tumor size and lymph node involvement.^8^ The PANAMA score was created using data from 2 high-volume centers with heterogeneous neoadjuvant treatment regimens. The validation of this score in a real-world multicenter setting of patients undergoing neoadjuvant treatment with the first-line treatment regimen FOLFIRINOX, including prediction of recurrence, is lacking.

Besides, the question of who benefits from adjuvant treatment after neoadjuvant treatment remains unanswered.^9^ One international study found that adjuvant therapy is only beneficial in pathologically node-positive patients,^10^ but this could not be confirmed by others.^11^ Therefore, a combination of variables, such as given in the PANAMA score, may be more adequate for such treatment decisions.

To guide postoperative patient counseling and adequately council patients on their prognosis, robust prognostic models are needed.^12^ Therefore, this multicenter study aimed to validate the prognostic value of the PANAMA score for overall survival (OS), and whether the PANAMA score can support clinical decision-making for adjuvant therapy in patients with resected PDAC following FOLFIRINOX.

## METHODS

### Study Design and Patient Cohort

This study represents a retrospective international multicenter study supported by the European-African Hepato-Pancreato-Biliary Association﻿. An existing cohort of patients after neoadjuvant treatment with FOLFIRINOX and resection for PDAC from 31 centers in 19 European countries was used to validate the PANAMA score.^10^ The study was approved by the scientific committee of the European-African Hepato-Pancreato-Biliary Association and complied with the “Strengthening the Reporting of Observational Studies in Epidemiology” guidelines, the “Transparent Reporting of a multivariable prediction model for Individual Prognosis or Diagnosis” statement, and with the 1964 Helsinki Declaration and its later amendments.^13,14^

All consecutive patients who underwent a pancreatoduodenectomy, left pancreatectomy, and total pancreatectomy for PDAC between 2012 and 2018 were screened for eligibility. Only patients who underwent at least 2 cycles of neoadjuvant treatment with FOLFIRINOX were included in the final analysis. A full dose FOLFIRINOX treatment cycle consisted of 5-fluorouracil (2400 mg/m^2^), leucovorin (400 mg/m^2^), irinotecan (150 or 180 mg/m^2^), and oxaliplatin (85 mg/m^2^) every 2 weeks.^10^ Modifications such as dose reductions did not lead to the exclusion of patients. Exclusion criteria encompassed patients with missing data for the components of the PANAMA score, metastatic disease at the time of resection, and patients without macroscopic complete resection of the tumor (R2 or exploration only). Furthermore, to reduce the bias of postoperative mortality to adjuvant treatment allocation, patients with in-hospital and 90-day mortality were excluded.^15^

### Definitions

Clinicopathologic data and survival outcomes were collected at each local institution. The National Comprehensive Cancer Network criteria were used for preoperative local tumor staging, whereas the American Joint Committee on Cancer (AJCC) Staging Manual eighth edition was used for postoperative staging.^16,17^ The PANAMA score was calculated according to the original publication.^8^ Table 1 shows the cutoffs used for the variables of the PANAMA score (pathologic tumor size, number of positive lymph nodes, resection margin status, and CA19-9 level). Risk groups were formed with 0 to 2 accumulated points categorized as low-risk, 3 to 5 as intermediate-risk and 6 to 8 points as high-risk for worse survival outcomes like the original publication (an online calculator can be found under the following domain: https://www.evidencio.com/models/show/10662?v=2.1). OS was defined as the time from surgery to death. Time to recurrence was defined as the period elapsed between surgery and the occurrence of local or distant recurrence. Patients were censored at the date of the last follow-up.

**TABLE 1:** Determinants of the PANAMA Score

Points	Tumor size (mm)	Positive nodes	Resection margin status*	CA19-9 after neoadjuvant Tx. (U/mL)
0	≤20	0–1	R0	≤37
1	21–30	2–3	—	>37
2	31–40	≥4	R1	—
3	>40	—	—	—

0–2 points = low-risk, 3–5 points = intermediate risk, 6–8 points = high-risk.

* Resection margin status: 1 mm rule, all margins and specimen surfaces. Tumor size and number of positive nodes after neoadjuvant chemotherapy in pathologic evaluation.

### Statistical Analysis

Statistical analysis was conducted using the R statistical software (version 4.2.3). Categorical data were summarized as frequencies and percentages, while continuous data were reported as mean with SD or median and interquartile ranges (IQRs). Comparisons of differences in the distribution of categorical data were done with the χ^2^ test, whereas the Mann-Whitney *U* test was used for continuous data. Any missing data were acknowledged in the baseline tables and excluded from group comparisons. Log-rank tests were used to determine the discriminatory value of the PANAMA and TNM scores. An additional analysis was performed to account for CA19-9 non-secretors (<5 U/mL). The predictive accuracy of the risk models was evaluated by the concordance index (c-index). A bootstrapping approach with 1000 iterations was applied to compare areas under the curve (AUCs) of the PANAMA and AJCC-TNM score for the prediction of survival rates every 12 months using time-dependent receiver-operating characteristic analysis. Hereby, AUCs were computed using the “timeROC” package with marginal weighting in a nonparametric approach. Mantel-Cox regression and Cox proportional hazard regression with interaction analysis were performed using the “Survival,” “Survminer,” and “Publish” packages. A landmark analysis after 18 months was performed to assess possible allocation bias to adjuvant treatment. Sensitivity analysis on the effect of adjuvant therapy on OS was performed on patients who underwent partial neoadjuvant treatment. According to Stoop et al,^18^ the cutoff of 8 cycles was defined as an acceptable length for complete neoadjuvant treatment. Data were then visualized with Kaplan-Meier curves and forest plots using “Ggplot2” and “Forester.” Hazard ratios (HRs) and 95% CIs were calculated for each assessed variable. A 2-sided *P* value of <0.05 was considered statistically significant.

## RESULTS

### Patient Cohort

Overall, 595 patients with resected PDAC following FOLFIRINOX treatment were identified. Patients with metastatic disease (n = 17), R2 resections (n = 19), postoperative in-hospital or 90-day mortality (n = 16), missing values for calculation of the PANAMA score, or missing follow-up (n = 160) were excluded. Hereafter, 383 patients remained available for the final analysis. Of those, 111 (36%) had resectable, 151 (49%) had borderline resectable, and 45 (15%) had locally advanced PDAC according to National Comprehensive Cancer Network (unknown n = 76). The median number of neoadjuvant cycles was 6 (IQR: 5–8). A dose reduction was reported in 90 patients (24%), and 57 patients (15%) were switched to another regimen during the neoadjuvant treatment phase. Additional neoadjuvant radiotherapy was administered in 97 (25%) patients. After neoadjuvant treatment, 20 (5%) patients had a complete radiologic response, 219 (58%) had a partial response, 138 (36%) had stable disease, and 3 (1%) had progressive disease according to the RECIST criteria. Pathologic complete response was observed in 26 (7%) patients. The median follow-up for patients alive at the last follow-up was 30.2 months (IQR: 21.0–39.5).

### PAncreatic NeoAdjuvant MAssachusetts Score and Survival

A total of 187 (49%) patients were low-risk according to the PANAMA score, 137 (36%) intermediate-risk, and 59 (15%) high-risk. Clinicopathologic characteristics are presented in Table 2. Of note, low-risk patients were more likely to have very low CA19-9 (<5 U/mL), received more cycles of neoadjuvant treatment, and had higher rates of additional neoadjuvant radiotherapy. Furthermore, less radiologic response was observed in the high-risk individuals who also had more extended resections, including vascular resections and total pancreatectomy.

**TABLE 2:** Clinicopathologic Characteristics of the PANAMA Risk Groups

PANAMA score risk groups	Low-risk (N = 187)^*^	Intermediate (N = 137)^*^	High-risk (N = 59)^*^	*P* ^†^
Sex (F)	91 (49)	70 (51)	22 (37)	0.195
ECOG performance status (0)	111 (65)	79 (66)	33 (75)	0.418
Unknown	15	17	15	—
NCCN stage				0.068
Resectable	51 (36)	46 (41)	14 (26)	—
Borderline	73 (51)	53 (48)	25 (47)	—
Locally advanced	19 (13)	12 (11)	14 (26)	—
Unknown	44	26	6	—
Radiologic response (RECIST)				<0.001
0: complete response	18 (9.7)	2 (1.5)	0	—
1: partial response	111 (60)	81 (60)	27 (47)	—
2: stable disease	57 (31)	50 (37)	31 (53)	—
3: progression	0	3 (2.2)	0	—
Unknown	1	1	1	—
Neoadjuvant FOLFIRINOX cycles	7.00 (2.88)	6.89 (3.84)	5.78 (2.48)	0.022
Unknown	1	2	0	—
Dose Reduction of neoadjuvant treatment	44 (24)	31 (23)	15 (25)	0.923
Unknown	2	1	0	—
Switch of neoadjuvant regimen	27 (14)	18 (13)	12 (20)	0.418
Receipt of neoadjuvant radiotherapy	63 (34)	28 (20)	6 (10)	<0.001
CA19-9 <5 U/mL	27 (14)	8 (6)	1 (2)	0.003
Operation type				<0.001
Distal pancreatectomy	25 (13)	19 (14)	8 (14)	—
Pancreatoduodenectomy	154 (82)	104 (76)	35 (59)	—
Total pancreatectomy	8 (4.3)	14 (10)	16 (27)	—
Venous resection	56 (30)	58 (42)	40 (68)	<0.001
Unknown	1	0	0	—
Arterial resection	6 (3.2)	5 (3.6)	16 (27)	<0.001
Postoperative complication rate	80 (43)	66 (48)	23 (39)	0.431
Adjuvant chemotherapy	118 (65)	100 (74)	39 (74)	0.188
Unknown	6	2	6	—
Type of adjuvant treatment				NA
FOLFIRINOX	25 (22)	21 (22)	12 (31)	—
Gemcitabine-capecitabine	5 (4.3)	2 (2.1)	9 (23)	—
Gemcitabine	68 (59)	53 (55)	11 (28)	—
Other	17 (15)	21 (22)	7 (18)	—
Unknown	72	40	20	—

^*^ n (%); mean (SD).

^†^ Pearson χ^2^ test; Kruskal-Wallis rank sum test; Fisher exact test.

NA indicates not available; NCCN, National Comprehensive Cancer Network.

**FIGURE 1:**
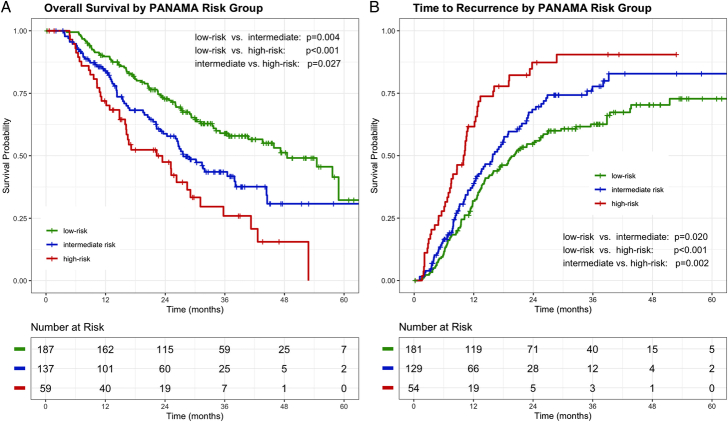
Kaplan-Meier curves for OS (A) and time to recurrence (B) stratified by PANAMA risk groups. Survival curves stratified by PANAMA risk groups showed differences in OS and time to recurrence. Censored patients are indicated by crosses. *P* values for group comparisons are calculated by log-rank test.

The PANAMA score showed moderate concordance with OS (c-index: 0.60). In addition, a stepwise reduction of median OS was observed per risk group [low-risk: 48.5 months [95% CI: 40.7–not reached (nr)], intermediate-risk: 27.6 months (95% CI: 24.3–38.1), and high-risk 22.3 months (95% CI: 16.1–31.1), Fig. 1A]. Similar results were observed for time to recurrence with a median of 20.1 months (95% CI: 15.9–26.0) in low-risk patients and 15.8 months (95% CI: 12.5–20.7) in intermediate-risk patients (*P* = 0.020). High-risk patients had the shortest median time to recurrence with 9.9 months (95% CI: 7.4–12.4), which was significantly shorter compared with low-risk patients (log-rank *P* < 0.001) and intermediate-risk patients (log-rank *P* = 0.002, Fig. 1B).

Exclusion of CA19-9 non-secretors (<5 U/mL) did not improve OS of the low-risk group [median ﻿OS (mOS)﻿: 48.5 months (95% CI: 40.7–nr)] or the model’s performance (c-index: 0.61, Supplemental Digital Content Fig. 1, http://links.lww.com/SLA/F397).

### Comparing PAncreatic NeoAdjuvant MAssachusetts and American Joint Committee on Cancer-TNM

**FIGURE 2:**
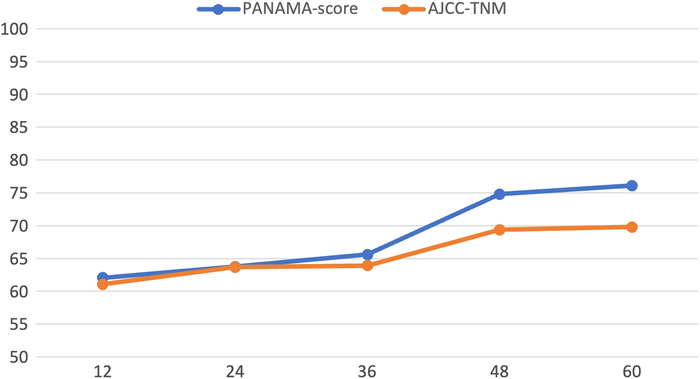
Comparisons PANAMA and AJCC-TNM AUC over time. AUC (*y*-axis) in percentage are shown over time (years, *x*-axis) after 1:1000 bootstrapping. Comparisons were not significant with *P* values of 0.746, 0.957, 0.576, 0.221, and 0.387 for 1, 2, 3, 4, and 5-year survival, respectively.

The AJCC-TNM staging system also had moderate concordance with OS (c-index: 0.60), but the differences between AJCC-stage 0 [mOS: 58.9 months (95% CI: 58.9–nr)] and AJCC-stage I [mOS: 44.9 months (95% CI: 34.2–nr), *P* = 0.078] and between AJCC-stage II [mOS: 31.6 months (95% CI: 26.0–43.6)] and AJCC-stage III [mOS: 22.2 months (95% CI: 16.2–28.4), *P* = 0.060] did not reach statistical significance (Supplemental Digital Content Fig. 2, http://links.lww.com/SLA/F397). Similar results were observed for time to recurrence (Supplemental Digital Content Fig. 3, http://links.lww.com/SLA/F397). After bootstrapping, the AUC of predicting 3-year survival was 0.66 (95% CI: 0.59–0.73) for the PANAMA score and 0.64 (95% CI: 0.57–0.71) for the AJCC-TNM score (*P* = 0.579). AUCs at other time points are shown in Figure 2.

### PAncreatic NeoAdjuvant MAssachusetts and Survival With Adjuvant Treatment

**FIGURE 3:**
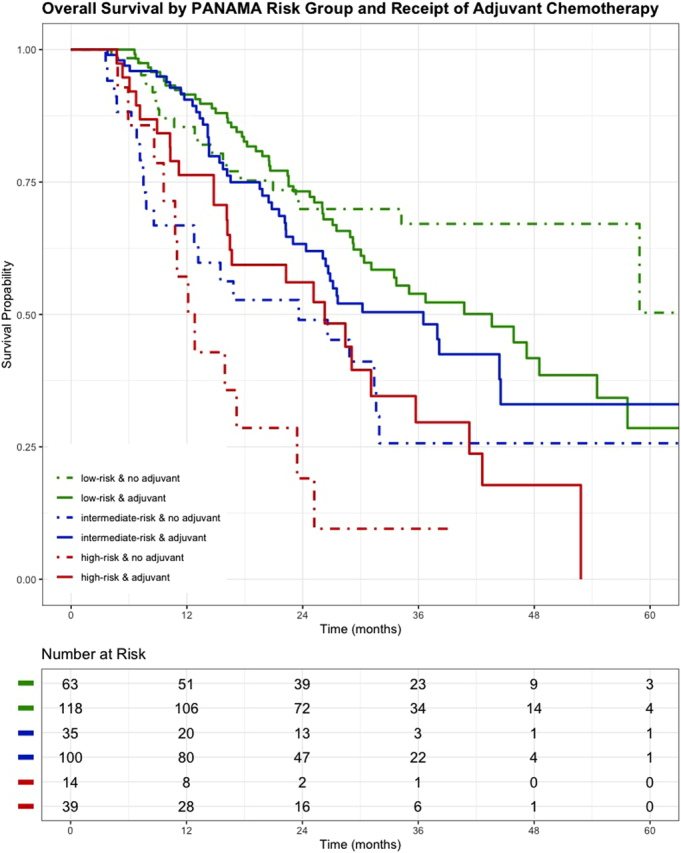
Kaplan-Meier curve for OS stratified by PANAMA risk groups and receipt of adjuvant treatment. No receipt of adjuvant therapy is indicated by dotted lines, whereas continuous lines indicate subroups that received adjuvant treatment. Median OS for low-risk [no adjuvant: nr (95% CI: 58.9–nr) vs adjuvant 43.6 months (95% CI: 31.1–57.7), log-rank *P* = 0.152], intermediate risk [no adjuvant: 23.6 months (95% CI: 12.8–nr) vs adjuvant 36.5 months (95% CI: 26.4–nr), log-rank *P* = 0.008], and high-risk [no adjuvant: 12.5 months (95% CI: 10.8–nr) vs adjuvant 26.3 months (95% CI: 16.5–42.6), log-rank *P* = 0.038]. C-index: 0.62.

In total, adjuvant therapy was administered in 257 (70%) patients with a median of 6 cycles (IQR: 4–6). According to PANAMA-risk groups, 118 patients (65%) low-risk, 100 (74%) intermediate-risk, and 39 (74%) high-risk group received adjuvant treatment (*P* = 0.188). In the PANAMA low-risk group, adjuvant treatment was not associated with a difference in OS (HR: 1.50, 95% CI: 0.92–2.50). In patients with intermediate-risk (HR: 0.58, 95% CI: 0.34–0.97) and high-risk (HR: 0.47, 95% CI: 0.24–0.94), adjuvant treatment was associated with improved OS (Fig. 3).

**FIGURE 4:**
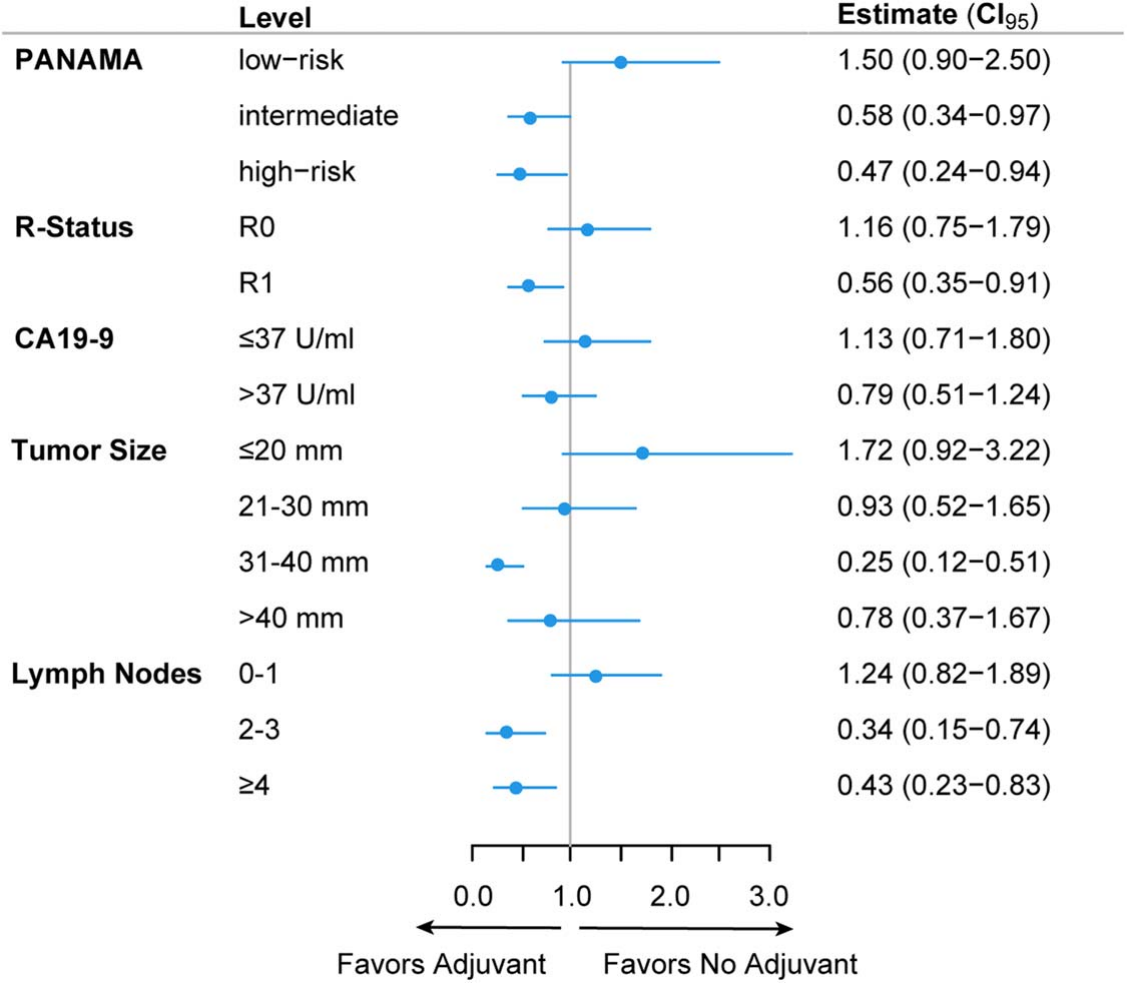
Treatment benefits derived from adjuvant chemotherapy. Subgroup analyses show different OS benefits associated with the receipt of adjuvant treatment. *P* interaction for differing treatment effects between subgroups: PANAMA score risk groups (*P* = 0.008), resection margin status (*P* = 0.030), pathologic tumor size (*P* = 0.013), lymph-node status (*P* = 0.003), and CA19-9 (*P* = 0.274).

Subgroups derived from PANAMA-risk points that did have an improved survival associated with additional adjuvant treatment after neoadjuvant FOLFIRINOX were those with a positive resection margin (R1: HR: 0.56, 95% CI: 0.35–0.91), those with a larger tumor size (31–40 mm: HR: 0.25, 95% CI: 0.12–0.51), and patients with node-positive disease (2–3 positive nodes: HR: 0.34, 95% CI: 0.15–0.74, and ≥4 nodes: HR: 0.43, 95% CI: 0.23–0.83, Fig. 4). Resection margin status (*P* interaction = 0.030), pathologic tumor size (* P* interaction = 0.013), and lymph-node status (*P* interaction = 0.003) were determinants associated with adjuvant therapy benefit, whereas no significant difference in treatment effect on OS was observed between the CA19-9 elevated and <37 U/mL group (*P* interaction = 0.274). Altogether, adjuvant therapy was associated with different treatment effects on OS when comparing the intermediate and high-risk group to the PANAMA low-risk group (*P* interaction = 0.008).

An 18-month landmark analysis showed significantly better OS of patients with PANAMA low-risk who did not receive adjuvant therapy compared with those who did undergo adjuvant treatment (log-rank *P* = 0.006), whereas there was no difference for the intermediate (log-rank *P* = 0.993), and high-risk groups (log-rank *P* = 0.508, Supplemental Digital Content Fig. 4, http://links.lww.com/SLA/F397).

Sensitivity analysis on patients with ≤8 cycles of neoadjuvant FOLFIRINOX [n = 268 (69%)], the PANAMA-score remained a significant determinant of improved survival associated with adjuvant treatment (*P* interaction = 0.033, Supplemental Digital Content Fig. 5, http://links.lww.com/SLA/F397).

## DISCUSSION

This multicenter study validated the discriminatory ability of the PANAMA score, as the low, intermediate, and high-risk groups were associated with OS and time to recurrence. Adjuvant therapy was not associated with a difference in OS in low-risk patients, whereas adjuvant treatment was associated with improved OS in intermediate-risk and high-risk patients.

Despite valid prognostic scores based on preoperative variables to inform surgical indications, postoperatively available variables with pathologic confirmation have demonstrated greater reliability in predicting prognosis after resection for PDAC.^8,12^ Accordingly, Leonhardt et al^19^ identified preoperative CA19-9, tumor size, nodal status, and tumor differentiation as key predictive factors for early recurrence after PDAC surgery. Furthermore, higher rates of early recurrence are also observed if patients do not receive adjuvant treatment.^19,20^ Since recurrence is the primary determinant of mortality in patients with resected PDAC, these factors warrant inclusion into a prognostic score. For example, the Amsterdam model includes margin status, tumor differentiation, lymph node ratio, and receipt of adjuvant therapy.^21^ However, the Amsterdam model was developed for patients undergoing upfront resection. A drawback, which is also relevant to the TNM staging system, is that neoadjuvant treatment effects may lead to tumor shrinkage and may transform positive to negative lymph nodes, therefore, not reflecting its original prognostic value.^4,22^ Although the treatment effect, as determined by pathologic treatment response, is prognostic following neoadjuvant treatment, the relevance of nodal negativity may, therefore, be decreased due to the possibility of undetected lymphatic metastasis.^23^ This indicates the need for additional parameters of minimal residual disease and the disseminative ability of tumor cells. In addition, due to the pathologic regression in most tumors, tumor grading is unreliable after neoadjuvant treatment.^22^ Moreover, including adjuvant treatment in a model does not improve postoperative patient counseling, as at that time point, this variable is unknown. This underscores the advantage of the PANAMA score, including variables that are known postoperatively and are widely available in clinical practice at the time the decision for adjuvant treatment should be made.

According to this analysis, the clinical adoption of the PANAMA score for the aim of prognostication for postoperative patient counseling is partly justified. Over time, the PANAMA score shows better predictive value for OS than the TNM-staging system. The differences for each time point, however, were insignificant. Furthermore, comparable to the AJCC-TNM staging system, only a moderate concordance with OS was observed. As indicated by Figure 2, the difference in the discriminatory ability of the PANAMA score compared with the AJCC-TNM staging system becomes more evident over time indicating some time-varying prognostic impact of the additional variables CA19-9 and margin status. Furthermore, the AUC for survival prediction models with classic clinicopathologic factors is low, making it difficult to reach statistical significance when comparing two scores with relevant overlap.^24^ Also, a cutoff for CA19-9 and inclusion of CA19-9 non-secretors is debatable but increases applicability to clinical practice. Nevertheless, these results imply that there is an urgent need for more reliable prognostic biomarkers beyond the classic clinicopathologic variables. To date, research on ctDNA and circulating tumor cells show promising results, but their clinical availability is not given, hindering their inclusion into multianalyte panels.^25,26^

Although increasing use of the neoadjuvant treatment approach is observed, many questions pertinent to its optimal application remain unanswered.^27^ First, the optimal number of cycles of neoadjuvant FOLFIRINOX is still a matter of debate. Additional cycles of neoadjuvant cytotoxic treatment may lead to lower rates of resection margin positive resections, smaller tumor sizes, and decrease in CA19-9, thus lowering PANAMA-risk score.^2,22^ Consequently, in this analysis, the number of cycles is inversely correlated with a lower PANAMA risk score. However, uncertainty remains if those with higher PANAMA-risk scores would profit from additional cycles and additional modalities to lower their risk for minimal residual disease. In this analysis, additional radiotherapy was associated with lower PANAMA risk scores and lower R1 rates (data not shown) but recently presented data from the randomized controlled PRODIGE-44 trial did not show an additional survival benefit or improved R0-rates when adding radiotherapy to FOLFIRINOX in the neoadjuvant setting.^28^ Furthermore, currently a total of 8 to 12 cycles is arbitrarily considered as complete treatment. This is derived from an arbitrary set treatment length of 6 months in initial studies of cytotoxic treatment for patients with metastatic PDAC, but more evidence is needed for the optimal treatment duration and sequencing in localized PDAC.^29^

Nevertheless, this gives rise to a second question: Is completion of chemotherapy up to 12 cycles associated with a survival benefit after neoadjuvant treatment? Hypothetically, patients with residual disease (ie, R1 resection as a surrogate marker) after resection would likely benefit from further cytotoxic treatment. In this study, margin status was indeed identified as a determinant of improved survival associated with adjuvant treatment as was advanced ypT-stage, which is a risk factor for local treatment failure. The most reliable surrogate marker for remaining systemic disease, the presence of more than one positive lymph node, was also identified as a significant determinant for adjuvant treatment benefit. Moreover, although the trend towards improved OS was not statistically significant, patients with elevated CA19-9 levels following neoadjuvant treatment exhibited a greater effect on OS from further adjuvant treatment than their respective counterparts with normal CA19-9 levels. In line with these findings, van Roessel et al^10^ found that only node-positive patients derive a benefit from further adjuvant treatment after neoadjuvant FOLFIRINOX (median cycles: 6, IQR: 5–8). These findings, however, were contradicted by another multicenter effort which also reported a benefit in node-negative patients.^11^ With a median of 2.7 cycles (IQR: 1.5–3.4) and predominantly gemcitabine-based regimens in the second study, the cohorts differed. Other retrospective studies showed that persistently elevated CA19-9, resection margin status, and larger tumor size predict an OS benefit from adjuvant therapy after neoadjuvant treatment.^9,30–34^ In an international multicenter study of 767 patients, Stoop et al^18^ showed an overall benefit of adjuvant treatment but no clinically relevant treatment effect for the subgroup of patients with ypN0, partial or complete radiologic response, or if patients were treated with ≥8 neoadjuvant cycles. Combining many of these biological variables into the PANAMA score revealed that patients with low-risk profiles may not derive a benefit from adjuvant treatment, whereas an association to improved survival associated with the receipt of adjuvant treatment is observed for patients with intermediate and high-risk profiles. This effect on OS was observed similarly in patients who underwent <8 neoadjuvant cycles, which underscores the importance of considering multiple factors for individualized treatment decisions versus completion in all patients. Overall, the PANAMA score has, therefore, been shown to be associated with different effects on OS stratified by receipt of adjuvant therapy after neoadjuvant treatment. Furthermore, the PANAMA score combines several predictive biomarkers associated with an OS advantage when undergoing further adjuvant treatment after neoadjuvant chemotherapy with FOLFIRINOX, thus mitigating the drawback of using a single biomarker for this important treatment decision.

The present study has several limitations. First, the retrospective design may introduce potential allocation bias. The varying practices regarding the preferred length of neoadjuvant treatment and the administration of adjuvant treatment among the contributing centers may have influenced the results and limited the overall generalizability of the findings. Nevertheless, it is possible that patients with similar characteristics would have been treated differently at the various centers, thus rendering this study a natural experiment.^35^ However, it is also likely that many treatment decisions were made on the basis of patient condition and preference. For instance, patients with a poor performance status may not receive all planned treatment cycles or the full dosage and may also be less likely to receive adjuvant treatment.^36^ Signs of this residual allocation bias are shown in the fast deviation of survival curves at the beginning of the follow-up period with a stable or decreasing difference after more than 12 months postoperatively. This observation was confirmed with an 18-month landmark analysis even showing longer OS in low-risk patients when they did not receive adjuvant therapy, whereas no association to differing OS was observed in intermediate and high-risk groups stratified by receipt of adjuvant therapy. Consequently, the effect of adjuvant treatment may be overestimated, and future randomized controlled trials must provide stronger evidence to inform individual treatment decisions on the optimal length and sequencing of cytotoxic treatment in patients with PDAC. Second, other determinants of a treatment benefit may be missing in the current analysis due to not being broadly available in clinical practice. For example, genetic subtyping has been shown to predict treatment benefits from neoadjuvant treatment, that is, the basal-like subtype derives more benefit as compared with the classic subtype.^37^ Furthermore, in the current study, adjuvant chemotherapy was not tailored to tumor characteristics and treatment response. Although a different regimen was used in most patients, statistical power was insufficient to perform a sensitivity analysis on the value of chemotherapy switch stratified by PANAMA risk groups. Translational studies show resistance development in organoids derived from patients after neoadjuvant FOLFIRINOX, suggesting that continuation of FOLFIRINOX therapy as an adjuvant treatment regimen may not be advantageous.^38^ In this study, in addition to the 15% of patients who received a change of regimen during the neoadjuvant course already, 78% of patients in the intermediate and 69% of patients in the high-risk groups were treated with an adjuvant regimen other than FOLFIRINOX, which may explain some of the increased efficacy of adjuvant treatment in these groups.^38^ Future studies need to confirm these results and investigate the value of regimen switch for adjuvant treatment after neoadjuvant FOLFIRINOX. Third, although significant discrimination for OS was shown between PANAMA-risk subgroups, only a moderate concordance can be achieved by clinicopathologic variables. Research and clinical introduction of more reliable biomarkers guiding patient prognostication and treatment decisions is urgently needed.^39^

## CONCLUSIONS

The PANAMA score, readily available through pancreascalculator.com, combines clinically available serologic and pathologic variables into a prognostic risk score that is successfully validated in this study for its discriminative ability in predicting OS and time to recurrence. Moreover, the PANAMA score is a reliable determinant of survival benefit associated with﻿ adjuvant therapy after neoadjuvant treatment. By combining clinically available predictive biomarkers of treatment benefit, it may be able to mitigate the drawbacks of using a single biomarker for this important treatment decision.

## Supplementary Material
