# Supplementary material for: Validation of the PANAMA Score for Survival and Benefit of Adjuvant Therapy in Patients With Resected Pancreatic Cancer after Neoadjuvant FOLFIRINOX
Source: Ann Surg. 2025 Jan 31;281(5):852–60. doi: 10.1097/SLA.0000000000006650 (PMC11974618; doi:10.1097/SLA.0000000000006650)

Supplemental Material

Supplemental Figure 1: Kaplan Meier Curve for Overall Survival Stratified by PANAMA Risk Groups after extclusion of CA19-9 non-secretors

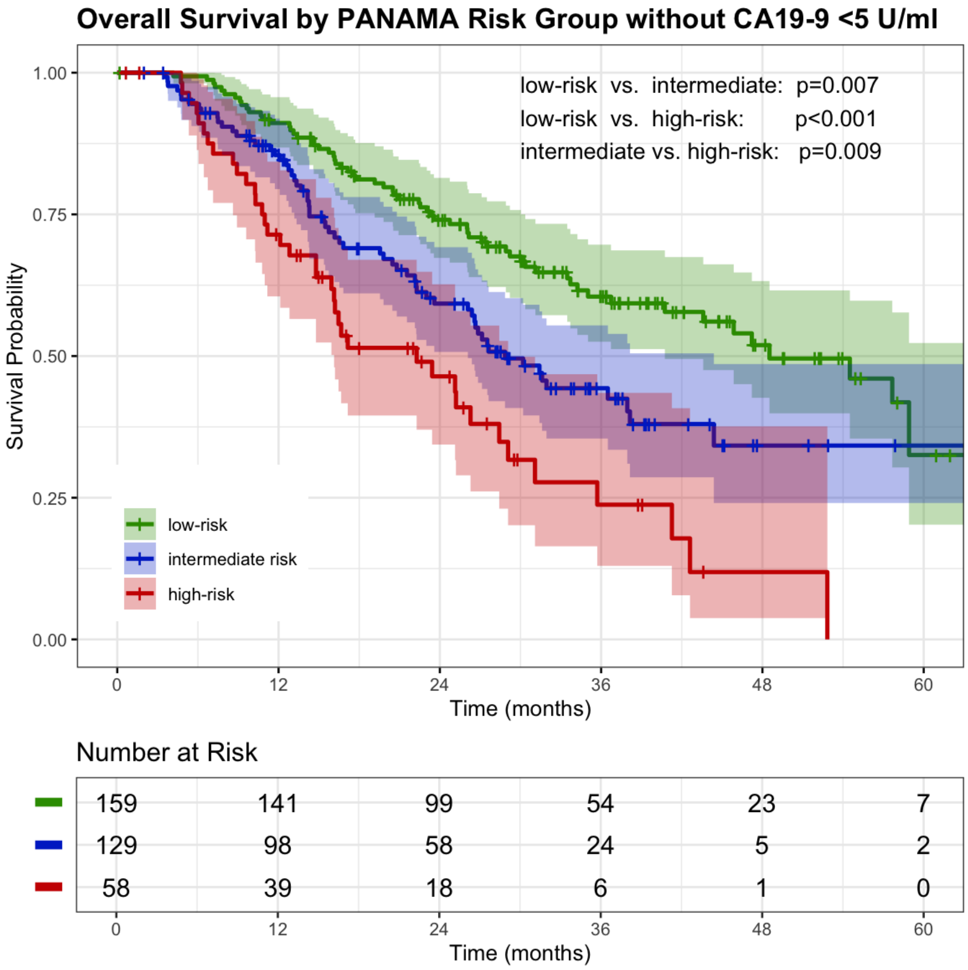

Legend: OS shown after exclusion of CA19-9 non-secretors (<5 U/ml). Median OS per PANAMA risk group: low-risk 48.5 months (95%CI:40.7-not reached), intermediate-risk 28.8 months (95%CI:26.1-44.4), and high-risk: 22.3 (95%CI:16.1-29.1). p values calculated by Log-Rank Test. Model performance c-index=0.61.

Supplemental Figure 2: Kaplan Meier Curve for Overall Survival Stratified by TNM-Stage

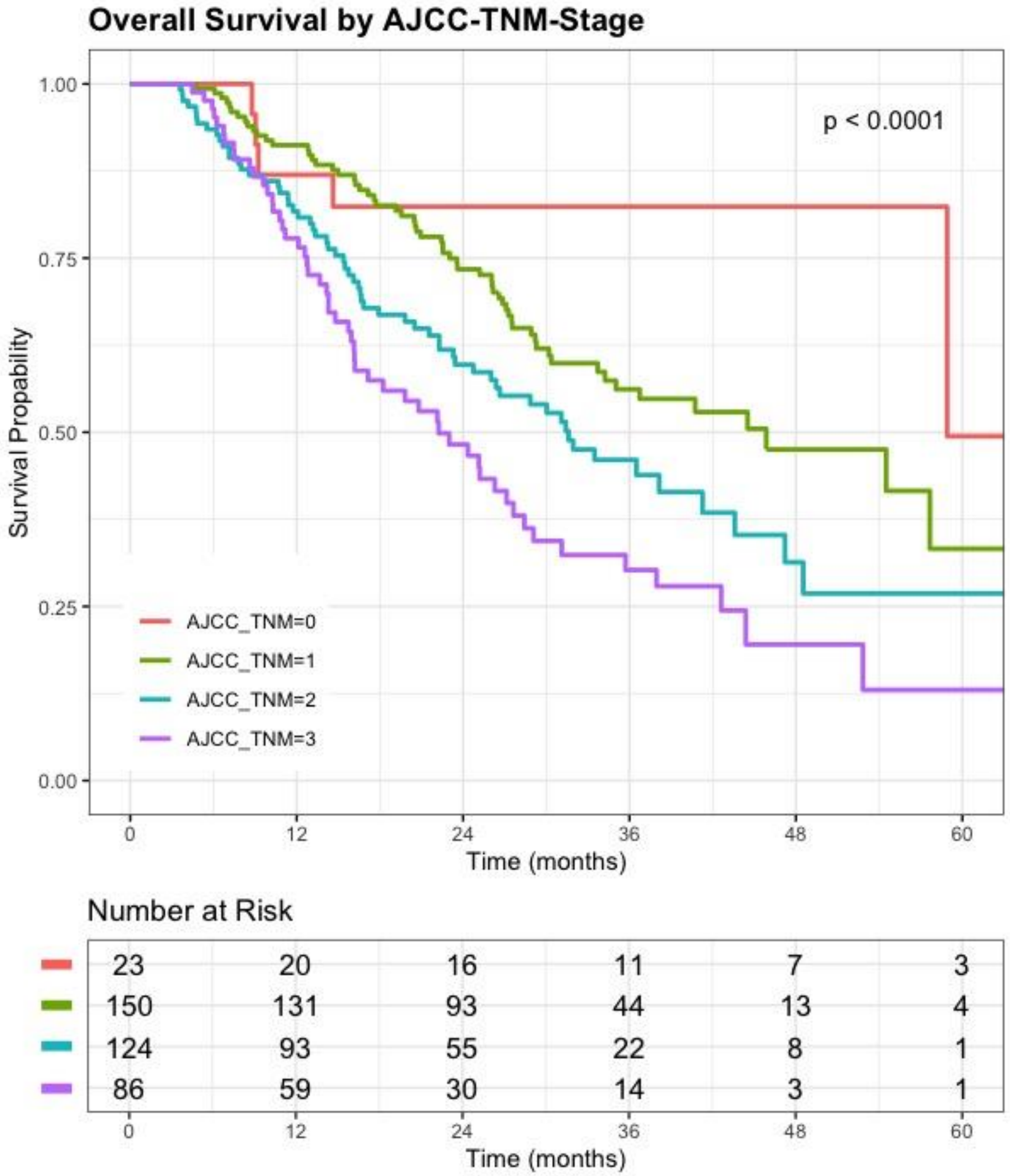

Supplemental Figure 3: Kaplan Meier Curve for Time to Recurrence Stratified by TNM-Stage

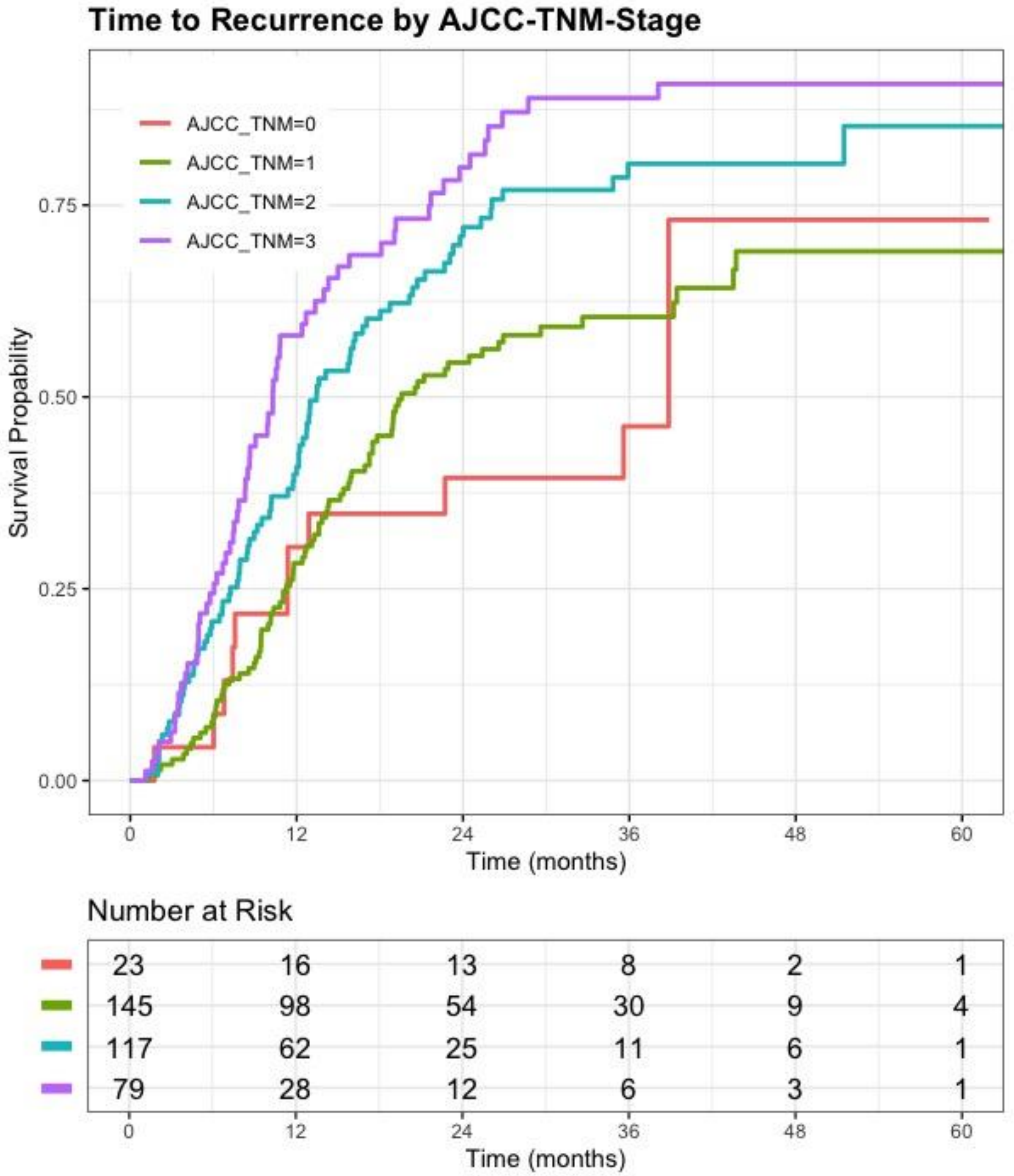

Figure Legend: Time to recurrence (TTR) is shown stratified by AJCC-TNM stage. No significant difference was observed between patients with complete response (TNM-0: mTTR 38.8, [95%CI:12.9-not reached]) and AJCC-TNM stage I (mTTR: 19.6 months, [95%CI:17.3-32.6], p=0.558) and for the comparison between AJCC-TNM stage II (mTTR 13.4 months [95%CI:12.2-18.0]) and AJCC Stage III (mTTR 10.3 months [95%CI:8.5-14.0], p=0.069). Concordance=0.60

Supplemental Figure 4: 18-Month Landmark Analysis for the Benefit of Adjuvant Treatment in PANAMA Subgroups

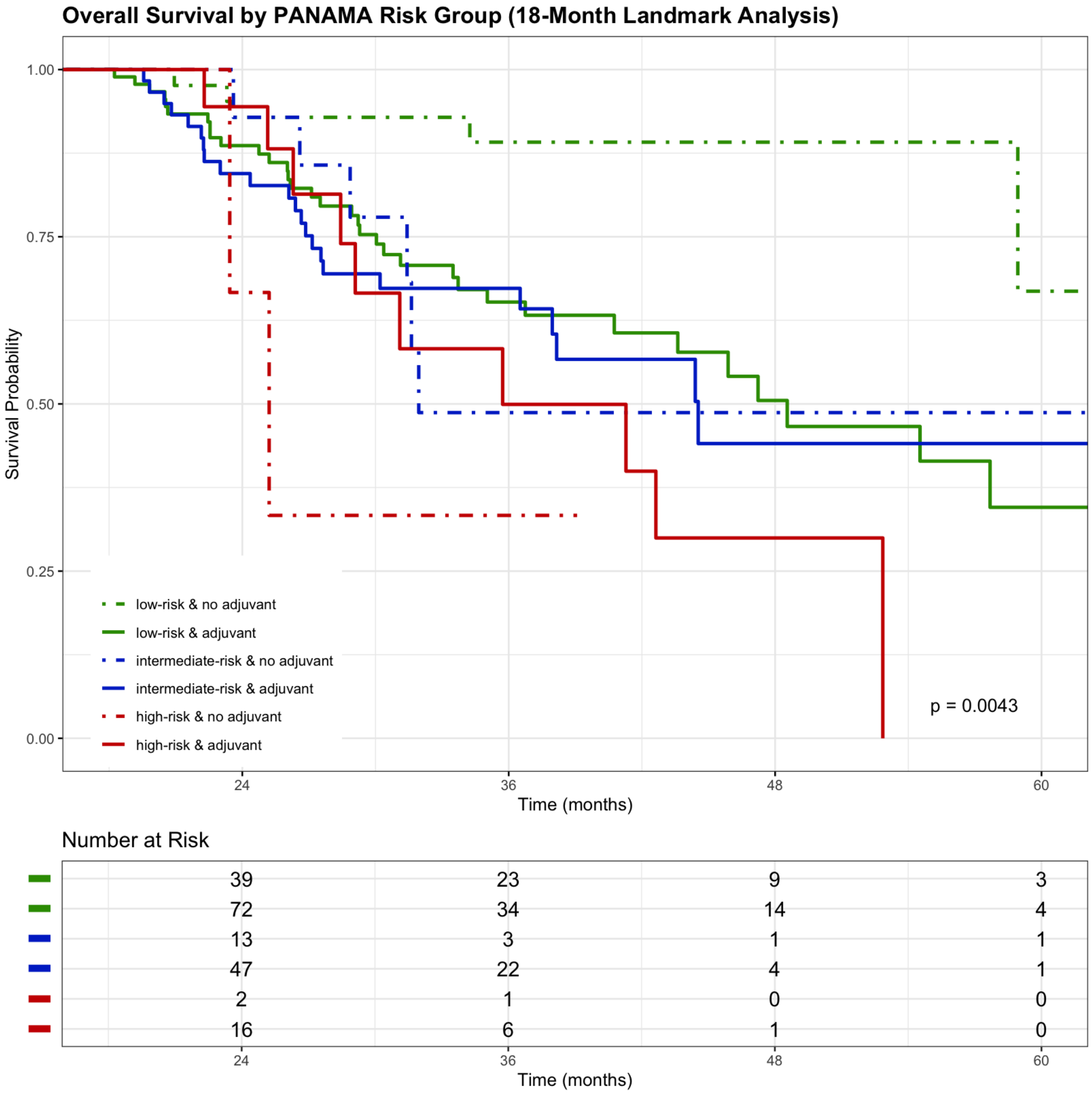

In those patients surviving at least 18-months, median OS per PANAMA risk-group and stratified by receipt of adjuvant therapy (C-index 0.60) was as follows

- Low risk: no adjuvant not reached (95%CI:58.9-nr) vs. adjuvant 48.5 months (95%CI: 40.8-nr), Log-Rank p=0.006)
- Intermediate risk: no adjuvant 31.9 months (95%CI: 31.4-nr) vs. adjuvant 44.5 months (95%CI:38.0-nr), Log-Rank p=0.993
- High-risk: no adjuvant 25.2 months (95%CI:23.4-nr) vs. adjuvant 35.7 months (95%CI: 29.1-nr) , Log-Rank p=0.508

Supplemental Figure 5: Treatment benefit derived from adjuvant chemotherapy with less than eight cycles of neoadjuvant treatment

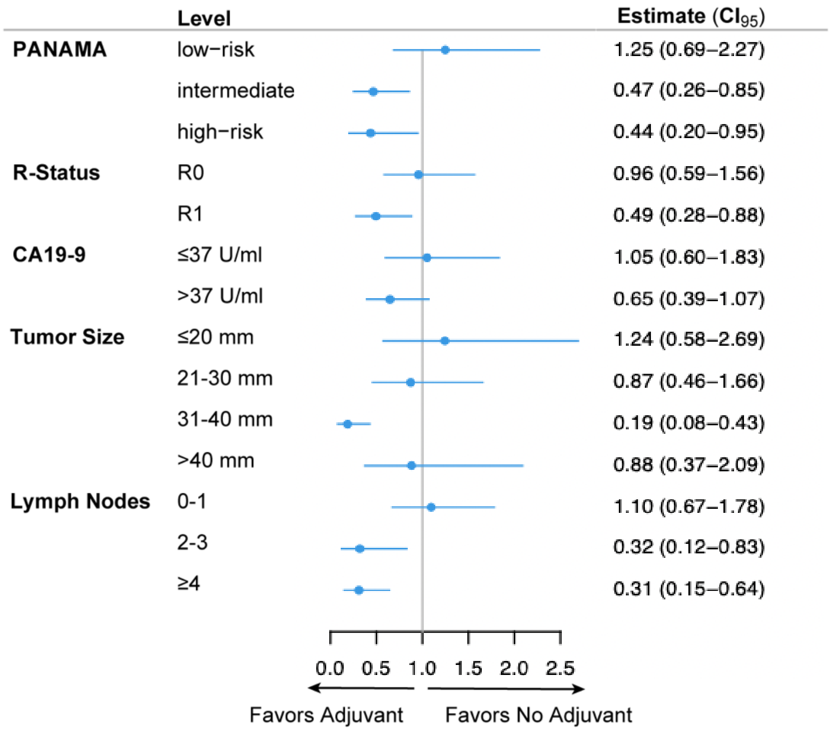

Supplement: Supplementary file 1 [file sla-281-852-s001.pdf]
